# Comparative transcriptome analysis, unfolding the pathways regulating the seed-size trait in cultivated lentil (*Lens culinaris* Medik.)

**DOI:** 10.3389/fgene.2022.942079

**Published:** 2022-08-10

**Authors:** Haragopal Dutta, Gyan P. Mishra, Muraleedhar S. Aski, Tejas C. Bosamia, Dwijesh C. Mishra, Jyotika Bhati, Subodh Kumar Sinha, Dunna Vijay, Manjunath Prasad C. T., Shouvik Das, Prashant Anupama-Mohan Pawar, Atul Kumar, Kuldeep Tripathi, Ranjeet Ranjan Kumar, Devendra Kumar Yadava, Shiv Kumar, Harsh Kumar Dikshit

**Affiliations:** ^1^ Division of Genetics, Indian Agricultural Research Institute, New Delhi, India; ^2^ Plant Omics Division, Central Salt and Marine Chemicals Research Institute, Bhavnagar, India; ^3^ Agricultural Bioinformatics, Indian Agricultural Statistics Research Institute, New Delhi, India; ^4^ ICAR-National Institute for Plant Biotechnology, New Delhi, India; ^5^ Division of Seed Science and Technology, Indian Agricultural Research Institute, New Delhi, India; ^6^ Laboratory of Plant Cell Wall Biology, Regional Centre for Biotechnology, Faridabad, India; ^7^ Germplasm Evaluation Division, National Bureau of Plant Genetic Resources, New Delhi, India; ^8^ Division of Biochemistry, Indian Agricultural Research Institute, New Delhi, India; ^9^ Division of Crop Science, Indian Council of Agricultural Research, New Delhi, India; ^10^ South Asia and China Program, International Center for Agricultural Research in the Dry Areas, NASC Complex, New Delhi, India

**Keywords:** RNA-seq, transcription factors, signal transduction pathway, *Lens culinaris*, seed parameters

## Abstract

Market class, cooking time, quality, and milled grain yield are largely influenced by the seed size and shape of the lentil (*Lens culinaris* Medik.); thus, they are considered to be important quality traits. To unfold the pathways regulating seed size in lentils, a transcriptomic approach was performed using large-seeded (L4602) and small-seeded (L830) genotypes. The study has generated nearly 375 million high-quality reads, of which 98.70% were properly aligned to the reference genome. Among biological replicates, very high similarity in fragments per kilobase of exon per million mapped fragments values (R > 0.9) showed the consistency of RNA-seq results. Various differentially expressed genes associated mainly with the hormone signaling and cell division pathways, transcription factors, kinases, etc. were identified as having a role in cell expansion and seed growth. A total of 106,996 unigenes were used for differential expression (DE) analysis. String analysis identified various modules having certain key proteins like Ser/Thr protein kinase, seed storage protein, DNA-binding protein, microtubule-associated protein, etc. In addition, some growth and cell division–related micro-RNAs like miR3457 (cell wall formation), miR1440 (cell proliferation and cell cycles), and miR1533 (biosynthesis of plant hormones) were identified as having a role in seed size determination. Using RNA-seq data, 5254 EST-SSR primers were generated as a source for future studies aiming for the identification of linked markers. *In silico* validation using Genevestigator^®^ was done for the Ser/Thr protein kinase, ethylene response factor, and Myb transcription factor genes. It is of interest that the xyloglucan endotransglucosylase gene was found differentially regulated, suggesting their role during seed development; however, at maturity, no significant differences were recorded for various cell wall parameters including cellulose, lignin, and xylose content. This is the first report on lentils that has unfolded the key seed size regulating pathways and unveiled a theoretical way for the development of lentil genotypes having customized seed sizes.

## Introduction

Lentil (*Lens culinaris* ssp. *culinaris* Medik.) or *Masur* (n = x = 7) is a self-pollinated, annual legume crop with a genome size of 4.2 Gbp (Arumuganathan and Earle, 1991; Dikshit et al., 2022). In 2019, the global lentil yield recorded was 5.73 million tons from a 4.8 million hectare area, and Canada was the largest producer (2.17 mt from 1.49 mha), followed by India (1.23 mt from 1.36 mha). Compared to the global productivity (1194.6 kg/ha), the lentil productivity of India is very poor (901 kg/ha) (FAO 2022). Madhya Pradesh and Uttar Pradesh are the largest lentil-producing states in India and are followed by West Bengal and Bihar (Mishra et al., 2022). For a very large section of the population residing mostly in developing countries, lentil is considered an inexpensive nutrition source, especially for proteins, carbohydrates, vitamins, dietary fibers, and micronutrients (Mishra et al., 2020a; Priti et al., 2021; Priti et al., 2022).

The lentil industry always tries to maintain the quality of the lentil, and seed size and shape is considered the key trait for obtaining the optimum quality. Cooking time and dehulling efficiency, which significantly govern the market preference are greatly influenced by the seed size and shape (Wang, 2008; Singh et al., 2022). Moreover, seed size and seed number govern the overall seed yield of any crop, which is mainly determined at the early seed developmental stages (Ruan et al., 2012; Mishra et al., 2020b) and is very precisely controlled by maternal and filial tissues (Weber et al., 2005). Advancements in understanding the seed development-associated pathway–related genes in lentils have been attributed to the availability of several markers, genomic resources, and cost-effective next–generation sequencing (NGS) tools. At present, the latest version (version 2) of lentil genome has been released (Ramsay et al., 2021). Thus, comparative RNA-seq analysis was aimed to identify the DEGs (differentially expressed genes) and pathways from the lentil genotypes differing significantly for the seed size during their early seed developmental stage.

Many reports demonstrating DE of various genes in the genotypes differing for the seed size in crops like peanut (Li et al., 2021), soybean (Du et al., 2017; Lu et al., 2017), etc. are available. Moreover, several complex regulatory networks including transcription factors, sugar, hormone signaling, and metabolic pathways regulating seed development are known in various plant species (Ruan et al., 2012; Du et al., 2017). In addition, molecular markers like EST-SSRs can be developed using the RNA seq data derived from the genotypes differing in the seed size. These novel markers can be used for the identification of linked markers using some mapping population for its application in the marker-assisted selection for seed size traits in lentils (Bosamia et al., 2015). Micro-RNAs (miRNAs) are the class of small noncoding RNAs (∼21-nucleotide long) that control the post-transcriptional expression of mRNA in lentils (Mishra et al., 2022). The miRNAs interact with the target mRNAs after getting loaded into the Ago-proteins (Dueck et al., 2012) and thus prevent gene expression (Winter et al., 2009). Using recent bioinformatics tools, putative miRNA target sites can be identified from the RNA-seq dataset, which will help in better understanding their role in the regulation of seed developmental pathways resulting in differential seed sizes in lentils.

The seed size is directly regulated by the chemical composition of cell wall in which the most distinctive constituent is cellulose (Costa and Plazanet, 2016), whereas lignin is the next most abundant biopolymer (Kathirselvam et al., 2019) and xylan is one of the highly abundant hemicelluloses (Scheller and Ulvskov, 2010). Thus, deciphering the complex cell wall dynamics associated with the regulation of seed size will help in understanding their relative function during seed development. Therefore, the RNA-seq approach was used for the identification of regulatory gene networks and pathways controlling seed size in lentils using genotypes differing significantly for the seed size.

## Materials and methods

### Plant materials, sampling, and seed parameter analysis using VideometerLab

**FIGURE 1:**
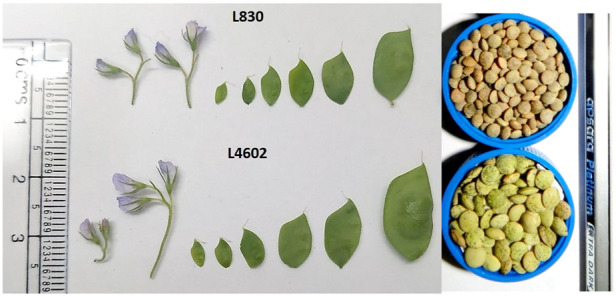
Different seed developmental stages and the developed seeds of L830 and L4602 genotypes.

**FIGURE 2:**
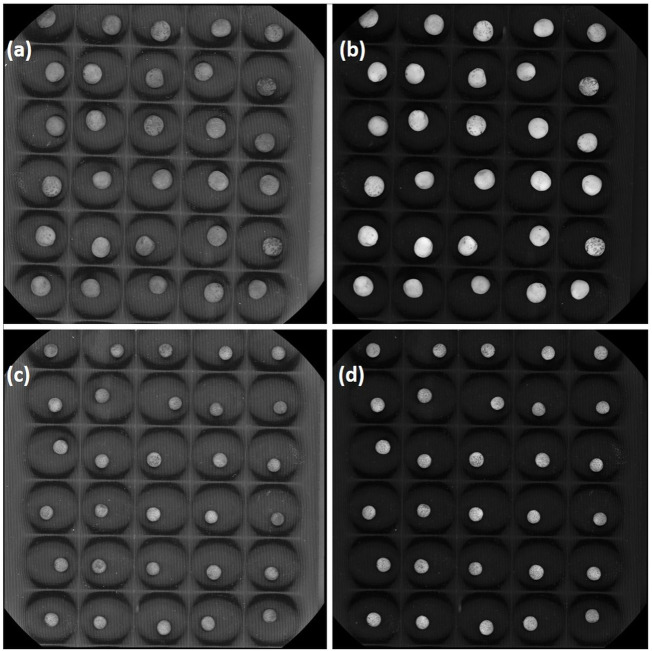
A representative mature seed photograph of lentil genotypes **(A,B)** L4602 and **(C,D)** L830 was taken at two different wavelengths [**(A,C)** at 375 nm; **(B,D)** at 590 nm] using VideometerLab.

Two lentil genotypes differing significantly in seed size, L830 (small-seeded; mean 1000 seed weight = 20.0g) and L4602 (large-seeded; mean 1000 seed weight = 42.13g) were used for the RNA-seq analysis (Figure 1; Table 1). Detailed seed phenotyping was done using the VideometerLab 4.0 instrument (Videometer A/S, Denmark), which acquires morphological and spectral information using strobed LED technology in the UV, visible, and NIR wavelengths (total 19 wavelengths; 365–970 nm) (Figure 2). The VideometerLab vision system was used to capture the images at 2056 × 2056 pixels of 30 seeds placed in special 3D printed plates, which were customized to fit our seed samples. The data generated were segmented, quantified, and plotted using custom-designed software (VideometerLab software ver. 2.13.83), which ultimately provided a vast array of information such as seed area, length, and width (Shrestha et al., 2015).

**TABLE 1:** Descriptive statistics for various seed traits of the genotypes L4602 and L830 using t-test.

S. No.	Variables	L4602		L830		Statistical significance
		Mean ± SD	SE mean	Mean ± SD	SE mean	
1	1000 seed weight (g)	42.13 ± 1.21	0.70	20.90 ± 1.82	1.10	HS
2	Area (mm^2^)	22.59 ± 1.17	0.262	11.02 ± 0.68	0.152	HS
3	Length (mm)	5.57 ± 0.169	0.038	3.82 ± 0.121	0.027	HS
4	Width (mm)	5.24 ± 0.179	0.04	3.71 ± 0.117	0.0262	HS
5	Width/Length	0.9399 ± 0.027	0.006	0.9723 ± 0.015	0.003	HS
6	Compactness (Circle)	0.9379 ± 0.025	0.006	0.9688 ± 0.014	0.003	HS
7	Compactness (Ellipse)	0.9983 ± 0.002	0.0003	0.999 ± 0.0004	0.00008	NS
8	Width/Area	0.2320 ± 0.006	0.001	0.3377 ± 0.011	0.002	HS
9	Volume (mm^3^)	0.007 ± 0.0002	0.00005	0.0048 ± 0.0001	0.00003	HS
10	Perimeter (mm)	15.477 ± 0.452	0.101	10.663 ± 0.369	0.082	HS

Where total count or replication (N) = 20; HS: Highly significant (*p* ≤ 0.01); NS, Not significant

The plants were grown in the National Phytotron Facility located at Indian Agricultural Research Institute, New Delhi, India (latitude: 28.6412°N; longitude: 77.1627°E; and altitude: 228.61-m AMSL) in 30 cm (diameter) pots (three seeds per pot) containing grow media consisted of coco peat: vermiculite: sand in 1:2:1 ratio. As we aimed to find the DEGs regulating the seed size at a very early stage, the flower buds were tagged on the day of fertilization and developing pods were collected 15 days after fertilization, and developing seeds were removed very carefully from the pods. These seed samples were then stored in RNAlater at −80°C before RNA extraction. A total of four samples were used which consisted of L830 (small-seeded) and L4602 (large-seeded) samples in two biological replications each.

### RNA extraction, cDNA library construction, and Illumina sequencing

As per the manufacturer’s protocol, total RNA was extracted from the seed samples of two lentil genotypes L830 and L4602 using the RNeasy plant mini kit (Qiagen, Hilden, Germany). DNAse I (Thermo, United States) treatment was given twice to remove the genomic DNA; afterward, RNA was dissolved in nuclease-free water. The RNA quality and integrity were measured using Bioanalyzer 2100 RNA 6000 Nano Kit (Agilent Technologies, Santa Clara, CA, United States) and on 2% agarose gel, respectively, and then high-quality RNA (1.5 µg; OD 260/280 = 2.0–2.1; OD 260/230 = ≥2.0–2.3; RIN value ≥ 7.0) were taken from two biological replicates (from two lentil genotypes) for the construction of four cDNA libraries using TruSeq mRNA Library Prep kit (Illumina Inc., United States). Using Illumina HiSeq 2500 platform 100 bp paired-end (PE) reads (100 × 2 = 200bp) were generated from the cDNA libraries at AgriGenome Labs Pvt. Ltd., Hyderabad, Telangana, India. The samples were labeled as L830 and L4602, in two replications (Rep-1 and Rep-2) each. The raw sequence data of these four PE libraries were submitted to the National Center for Biotechnology Information SRA database (Accession number PRJNA800200).

### Transcriptome assembly and annotation, and GO

FastQC (www.bioinformatics.babraham.ac.uk/projects/fastqc) was used for the analysis of the raw reads, whereas AdapterRemoval2 (ver. 2.2.0; https://github.com/MikkelSchubert/adapterremoval) was used to trim the low-quality bases (Phred score ≤ 30) and adapter sequences. Bowtie2-based SILVA database was used for the removal of rRNA sequences, and then, GC content, Q30, and total clean reads were determined in the high-quality reads (HQRs). For the alignment of trimmed reads, Bowtie2 ver. 2.2.2.9 (http://Bowtie2-bio.sourceforge.net/index.shtml) was used and unigenes with ≥200 bp transcript length were assembled for the estimation of transcript expression and their downstream annotations. For alignment, the lentil reference genome (Ver. 2) was used (Ramsay et al., 2021). Read count normalization was done, and transcript expression levels were determined using FPKM (fragments per kilobase of exon per million mapped fragments) analysis. The annotation of assembled unigenes was done using UniProt Plant Database and Gene Ontology (GO) terms were identified using BLAST2GO (https://www.blast2go.com; Conesa et al., 2005).

### Differential gene expression and GO enrichment analysis

The normalization of gene expression values was done using FPKM and fold change (FC), whereas expression was calculated using Cuffdiff (http://cole-trapnelllab.github.io/cufflinks/cuffdiff/). Differential gene expression analysis was performed by considering the individual transcript expression level counts in small- (L830) and large-seeded (L4602) samples using the edgeR (http://www.bioconductor.org/packages/2.12/bioc/html/edgeR.html) program (Robinson et al., 2010). The false discovery rate (FDR ≤ 0.05) and Log2 Fold Change (Log2FC +2/−2) were used as a threshold for the identification of DEGs as upregulated or downregulated. The GO terms for all assembled unigenes were extracted wherever possible. “Goseq” of “R package” (https://bioconductor.org/packages/release/bioc/html/goseq.html) was used for the GO enrichment analysis, and the identified GO terms were grouped into biological process, cellular component, and molecular function categories. Afterward, a scatter plot was generated and visualized for the GO-enriched terms using Revigo (http://revigo.irb.h) (Supek et al., 2011). The information available in the Kyoto Encyclopedia of Genes and Genomes (KEGG) Automatic Annotation Server (http://www.genome.jp/kegg/) database was used to perform the metabolic pathway analysis of the assembled unigenes (Kanehisa and Goto, 2000).

### miRNA and miRNA target sites identification

Precursor miRNA sequences were identified using C-mii software, by searching the sequences against the miRbase database and identifying the hairpin-like secondary structures (Numnark et al., 2012). Then, putative miRNAs were identified having parameters like 1) predicted mature miRNAs length should be 18–24 nt; 2) a maximum of two mismatches over known mature miRNAs; 3) localization of mature miRNA in one arm of the predicted stem-loop structure; 4) not >5 mismatches between miRNA and guide miRNA sequence in the stem-loop structure; 5) presence of 30–70% A + U content in the miRNAs; 6) and a highly negative minimal folding free energy and minimal free energy index value of the secondary structure. Putative target genes were identified using psRNATarget (https://www.zhaolab.org/psRNATarget/) against the available *Arabidopsis thaliana* annotated data to understand the biological functions of the miRNAs (Dai et al., 2018) and miRNA target network was built using Cytoscape software (Shannon et al., 2003).

### Protein–protein interaction network

String network analysis was performed to understand the PPI network of the identified DEGs. The identified target list was first uploaded into the STRING database (https://string-db.org/) (Szklarczyk et al., 2015); afterward, highly interconnected proteins were identified using the MCODE plugin of Cytoscape v3.7.0 (Shannon et al., 2003). The final prediction of the PPI networks was based on *Medicago truncatula* as this showed very high sequence similarity.

### *In silico* validation analysis using Genevestigator®

The DEGs were filtered based on growth- and development-related GO terms and were then narrowed down as per their FDR value (<0.05). Peptide sequences of the filtered DEGs were extracted from the Uniprot database using BLASTP. The associated reference genes having maximum stability of expression against *Medicago truncatula* were identified by submitting the shortlisted target accessions (unique) as input in the Genevestigator^®^ software (Zimmermann et al., 2004). For the grouping of target genes having similar profiles across the above conditions, a hierarchical clustering tool was used and a Log2 ratio change value was obtained for the identified genes and the top 15 perturbations were presented as a heat map.

### SSR prediction and EST-SSR identification

The unigenes from present RNA-seq data were used for the SSR prediction using the MIcroSAtellite Identification Tool (MISA) (Thiel et al., 2003) and the development of genic-SSR markers. To avoid any already known SSR primers, primer sequences already available in the public domain were searched and unigenes were removed from our RNA-seq data using an in-house Perl script (Bosamia et al., 2015). Standalone primer3 ver. 0.4.0 tool was used for primer designing as per the default parameters (Untergasser et al., 2012). Furthermore, a set of developmental process–related SSRs were siphoned considering the GO terms associated with various developmental processes like embryo development, flower development, endosperm development, cell cycle process, seed growth, and reproductive shoot system development.

### Preparation of alcohol insoluble residue sample

The lentil seeds (600 mg) were crushed, flash frozen in liquid nitrogen, and then ground in Qiagen TissueLyser II (at 30 Hz for 2–3 min) to a fine powder. The 100-mg fine powder was incubated in 5 ml of 80% ethanol containing 4.0 mM HEPES buffer (pH 7.5) at 70°C for 30 min, cooled on ice, and centrifuged at 10000 rpm for 15 min. The supernatant was discarded and the residue was washed with 5 ml of 70% ethanol. The residue was further suspended in 5 ml of chloroform: methanol (1:1) for 3 min at room temperature and centrifuged at 14000 rpm for 15 min. The residue was washed with 5-ml acetone, and the pellet was dried in the desiccator and used as an AIR sample for further analysis (Pawar et al., 2017).

### Cellulose estimation using the Updegraff method

The Updegraff reagent (acetic acid: nitric acid: water = 8:1:2 v/v) was added to the 2 mg of AIR and incubated at 100°C for 30 min. The mixture was centrifuged (10,000 rpm; 15 min), washed with acetone four times, and the residue was dried overnight. The residue was hydrolyzed in 72% sulphuric acid and glucose was analyzed using anthrone assay (Updegraff 1969). A glucose standard curve was used to calculate the cellulose content.

### Estimation of xylose and *O*-acetyl content

A total of 2 mg of AIR was incubated with HCl and NaOH samples for xylose and acetyl content analysis, respectively. The mixture was neutralized with NaOH and HCl, respectively. The xylose and *O*-acetyl content were analyzed using Megazyme K-ACET and K-XYLOSE kits (Rastogi et al., 2022).

### Acetyl bromide soluble lignin content

The acetyl bromide solution (25%), which was diluted using acetic acid was incubated at 50°C for 2 h. The solubilized powder was then mixed with NaOH and hydroxylamine hydrochloride. The absorbance was taken at 280 nm, and lignin content was measured as explained in Foster et al. (2010).

### Lignin and cellulose estimation through Fourier transform-infrared spectroscopy

The fine lentil seed powder was analyzed for lignin and cellulose content using FT-IR spectroscopy as previously described (Pawar et al., 2017). In a word, a Tensor FT-IR spectrometer (Bruker Optics) equipped with a single-reflectance horizontal ATR cell (ZnSe Optical Crystal, Bruker Optics) was used for the analysis. The spectrum was taken within a range of 600 cm^−1^ to 4000 cm^−1^ with a resolution of 4 cm^−1^. The standard was prepared with potassium bromide powder. Each sample was measured twice (by removing and putting different aliquots of powder to evaluate their heterogeneity), and each spectrum was the average of 16 scans (Labbe et al., 2005; Canteri et al., 2019).

### Statistical analysis

Two sample *t*-test was performed to determine the genotypic variance (among L830 and L4602 genotypes) for the traits such as seed area, length, width, width/length ratio, compactness, width/area ratio, volume, perimeter, lignin, cellulose, and xylose contents. All statistical analyses were conducted using Minitab ver.18 software (Minitab 2020).

## Results

### Seed parameters

The lentil genotypes L4602 (42.13 g/1000 seeds) and L830 (20.90 g/1000 seeds) selected for the study differed significantly for 1000 seed weight. In addition, many other seed morphological traits were measured using the VideometerLab instrument (like area, length, width, width/length, compactness, width/area, volume, and perimeter) and showed significant variations for these genotypes (Table 1). It is of interest that the mean seed area (mm^2^), length (mm), and width (mm) of the genotypes L4602 (22.59, 5.57, 5.24, respectively) and L830 (11.02, 3.82, 3.71, respectively) were found significantly different between these genotypes (Table 1).

### RNA sequencing and reference transcriptome assembly

The developing seeds (15 days after pollination) were used for the isolation of RNA from both L830 and L4602 lentil genotypes for analyzing the transcriptional changes during the early seed development stage using the Illumina HiSeq 2500/4000 platform. The samples were named L830-Rep1, L830-Rep2, L4602-Rep1, and L4602-Rep2. More than 448 million reads have been generated from all four sample combinations, having an average GC content of 47.17%. The raw sequence ranged from 81.83 to 127.13 million (for L830 Rep1 and Rep2) and 120.56 to 118.93 million (for L4602 Rep1 and Rep2), respectively. After removal of adapters and rRNA sequences and passing through the low-quality reads nearly 375 million HQRs were obtained having a Phred score of ≥30. The clean reads ranged from 80.01 million to 99.4 million for L830 and from 78.74 million to 117.18 million for L4602 genotypes with an average Q30 quality score of 96.75%. The summary of different RNA-seq parameters of the studied genotypes is presented in Table 2.

**TABLE 2:** Summary of RNA-seq data in four developing seeds samples of lentils (small- and large-seeded).

Sample name/Attributes	L830-Rep1	L830-Rep2	L4602-Rep1	L4602-Rep2
Mean read quality (Phred score)	39.06	39.32	39.68	39.03
Number of raw reads (PE)	63569309	40918140	60281921	59467429
Number of bases (Gb)	12.71	8.18	12.05	11.89
GC (%)	46.34	46.24	48.02	48.08
% data ≥ Q30	95.39	95.83	97.09	95.02
Read length (bp)	100 × 2	100 × 2	100 × 2	100 × 2
Total read count	127138618	81836280	120563842	118934858
Clean read count	99405074	80015960	78742292	117185022
QC Pass (%)	78.19	97.78	65.31	98.53
Aligned Read Count	94320301	77115384	59243198	112377029
Aligned (%)	94.88	96.38	75.24	95.9

Where Rep1 and Rep2 are the two biological replications.

The preprocessed and rRNA removed reads were used for reference-based pair–wise alignment and assembly of HQRs with lentil genome using the Trinity program. From the filtered reads, ∼98.70% of reads from each sample were properly aligned back to the assembled unigenes, indicating HQR data for the assembly. The total number of PE reads obtained in the small-seeded (L830) sample ranged from 40.91 million to 63.56 million, whereas in the large-seeded (L4602) sample from 59.46 million to 60.28 million. All the assembled transcripts were ≥200bp long with a GC content of 46.24–48.08% (Table 2). The details of transcript length, FPKM range, and GC range are provided in Supplementary Material S1 and Supplementary Table S1.

### Differential gene expression analysis and transcript annotation

EdgeR program was used to find the total number of genes expressed in small-seeded (L830) and large-seeded (L4602) genotypes. Out of 512 identified DEGs, 199 showed upregulation and 313 downregulation (Supplementary Table S2). The assembly has generated 120,149 transcripts, and the longest transcript is of 32,136 bp size. A total of 43,369 unigenes were generated, of which 21,038 unigenes showed at least one significant hit in UniProt Plant Protein Database. The assembled unigenes were annotated using UniProt Plant Database for the identification of lentil gene function using the BLASTX program and 106,996 unigenes were considered for differential gene expression analysis at the threshold level [FDR≤ 0.05; Log2FC +2/−2] (Table 3, Supplementary Table S3). Correlation analysis was done using FPKM values of the two biological replicates of lentil samples and the very high similarity (R > 0.9) verified the consistency among the samples (Supplementary Table S1). The most similar BLASTx matches were recorded for *Medicago truncatula* (12101) followed by *Cicer arietinum* (6470), *Pisum sativum* (638), and *Glycine max* (178) (Supplementary Table S4), and the top 20 BLASTX hits are presented in Supplementary Material S1.

**TABLE 3:** Assembled transcript summary.

Annotation Category	Numbers
No. of assembled transcripts (Total: 120,149)	106,996
Longest Read length (bp)	32,136
Mean GC of transcripts (%) (All Assembled Transcripts: 38.96%)	38.83
Total No. of Unigenes considered for BlastX	43,369
No. of Unigenes with a hit in UniProt Plant Database	38,154
No. of Unigenes with a significant hit in UniProt Plant Database	21,038
No. of Unigenes with a significant hit in PMN Database	5,334

### GO enrichment, scatter-plot, and KEGG pathway analysis

**FIGURE 3:**
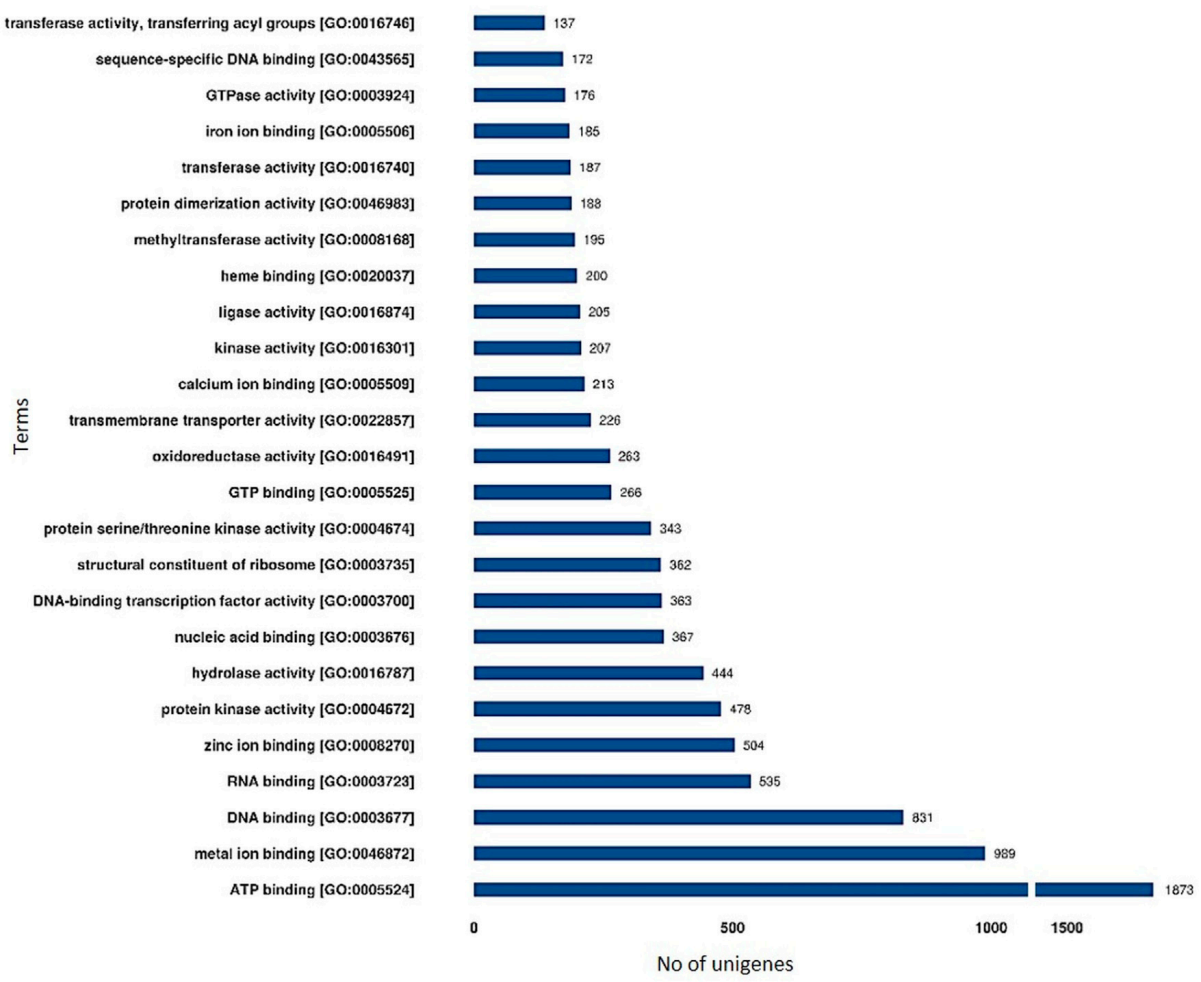
Functional annotation of unigenes based on Gene Ontology (GO) categorization of lentil genotypes differing for seed sizes (molecular function). These GO terms are classified into three categories (cellular component, molecular function, and biological processes).

The GO terms for all the assembled unigenes were extracted and grouped into biological process (1301), cellular component (403), and molecular function (1521) categories (Supplementary Table S5) using the Blast2GO program (http://www.blast2go.com/). For cellular component, the key enriched GO terms include “integral component of membrane,” “nucleus,” cytoplasm, “ribosome” etc., whereas for Molecular Functions these include “ATP, metal ion, DNA, and RNA binding,” “serine/threonine-protein kinase activity,” “hydrolase activity,” and transferase activity. DEGs were found enriched for several Biological Processes GO terms like “regulation of transcription,” “translation,” “protein folding and glycosylation,” “DNA replication,” “auxin activated signaling pathway,” “metal ion transport,” and “photosynthesis” (Figure 3; Supplementary Material S1). These signify the presence of substantial differences between large- and small-seeded lentil genotypes during the early seed development stage and also the vital role of identified DEGs resulting in differential seed size.

**FIGURE 4:**
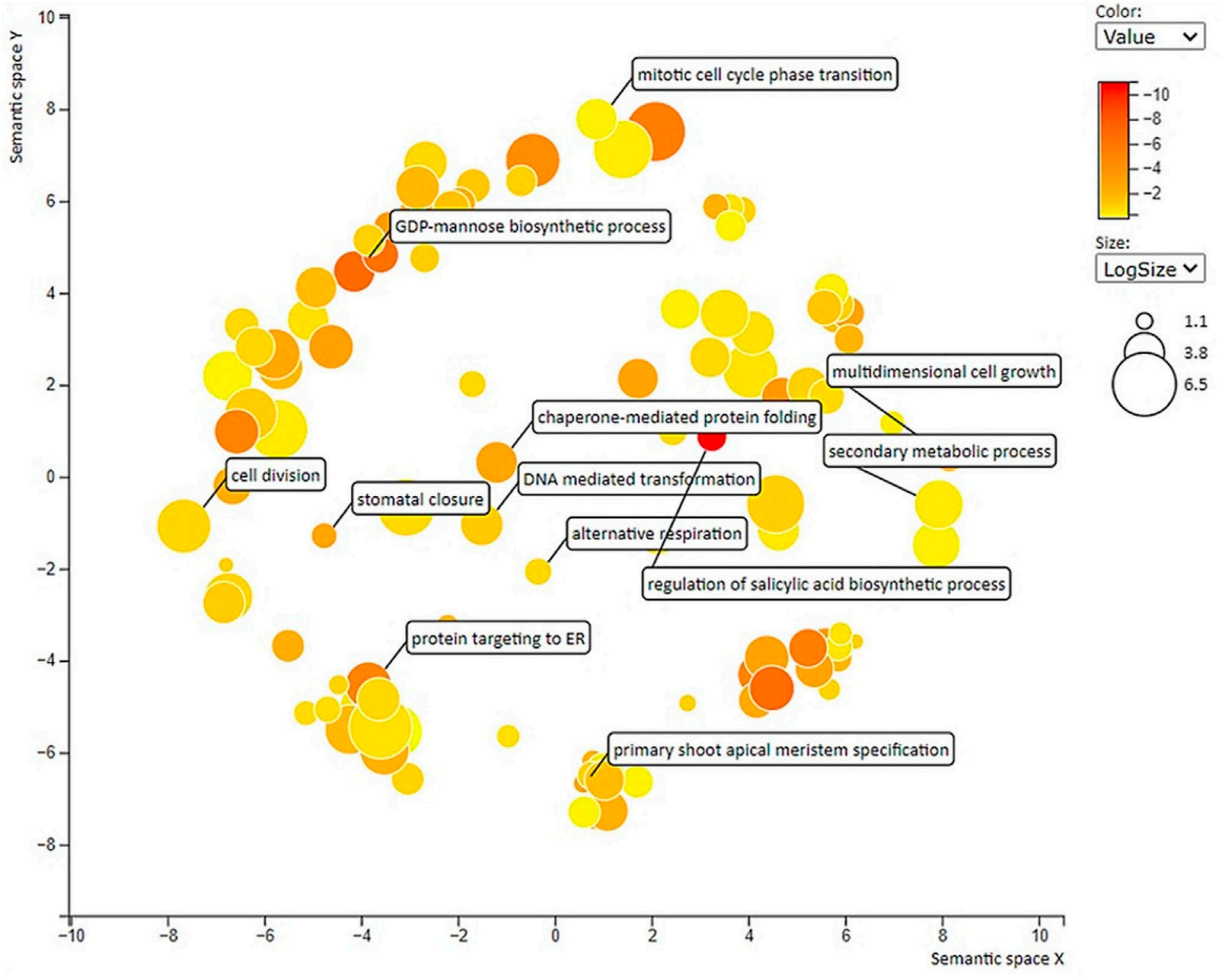
Scatter plot showing overrepresented GO term (p < 0.01) in all comparisons with labels having seed-size responsive terms. Different shades in circles indicate the difference in *p*-values (as given in the scale), whereas the bubble size indicates the frequency of the GO term.

All the DEGs with GO allotted [3224 Nos] were subjected to gene enrichment analysis and the enriched GO terms are further visualized as a scatter plot (Figure 4). Among various GO terms, “cell division” [GO:0051301], mitotic cell cycle phase transition [GO:0000278], multidimensional cell growth [GO:0009825], and “GDP mannose biosynthetic process” [GO: 0009298] are significantly enriched. To identify the major biological pathways which got altered in lentils when studied in large- and small-seeded genotypes at early seed development stage, the core DEGs were mapped to the KEGG pathways database. The KEGG annotated unigenes [13133 Nos] were found distributed to 25 KEGG pathways and the overrepresented pathways include: “glycolysis/gluconeogenesis,” “glyoxylate and dicarboxylate metabolism,” “pentose and glucuronate interconversions,” “pyruvate metabolism,” and “cyanoamino acid metabolism” (Supplementary Tables S6, S7). The details of the upregulated and downregulated KEGG enriched terms (biological process, cellular component, and molecular function) are presented as a pie chart (Supplementary Material S1).

### miRNA prediction, miRNA target site identification, and function analysis

Through homology searches of the sequences and miRNA prediction with the c-mii program, we identified 46,195 upregulated and 48,911 downregulated miRNA candidates (Supplementary Table S8). The putative miRNAs varied from 19 to 26 nt in length, with a majority of them being 21 nt in length. Furthermore, based on the multiplicity (≥10 numbers) of the miRNAs identified the key miRNAs identified from upregulated (miR838, miR1440, miR1533, miR3457, miR3637, miR3951, and miR6024) and downregulated DEGs (miR-190, miR902, miR1533, miR3437, miR3520, miR4353, miR5543, miR5576, miR5658, miR5720, miR7511, miR7743, and miR9742) are presented in Table 4. In upregulated category, the esi-miR3457-3p family was the largest family with nearly 400 members followed by gma-miR1533 (256), vvi-miR3637-5p (121), and aly-miR838-3p (100), csi-miR3951 (100), osa-miR1440b (100), sly-miR6024 (100), and stu-miR6024-3p (100). Likewise, from the downregulated DEGs the gma-miR4353 family was the largest (1225) followed by bdi-miR7743-3p (1089), ghr-miR7511 (625), osa-miR5543 (484), ppt-miR902i-3p (441), hme-miR-190 (256), and aly-miR3437-3p (154) (Supplementary Table S8). Among these, a few are growth and cell division–related miRNAs like miR3457 (cell wall formation), miR1440 (cell proliferation and cell cycles), and miR1533 (biosynthesis of plant hormones). This study for the first time could identify the transcriptome-based miRNAs and their targets from the RNA-seq data of two lentil genotypes differing in seed size.

**TABLE 4:** List of miRNAs and the target fragment identified from the RNA-seq data generated for seed size variations.

S. No.	miRNA identified	Target details	miRNA aligned fragment	Target aligned fragment	Multiplicity
**(a) From downregulated DEGs**					
1	gma-miR4353	Lcu.2RBY.Chr1:20126119-20138212	CAA​GUC​GUA​GCC​GGU​GUU​AUU​ACU	AGA​CAA​GAC​ACC​AGC​UGC​GAC​UUG	35
2	bdi-miR7743-3p	Lcu.2RBY.Chr7:474327216-474368813	UUU​GAA​CUU​UUG​UAU​UGG​AUC​UUU	CUA​GCU​CUA​AUA​CAA​AAG​UAU​GAA	33
3	ghr-miR7511	Lcu.2RBY.Chr7:474327216-474368813	AGA​AGU​UUU​GCA​UGU​GUA​GCU​GAG	CAU​AAC​UUC​UAA​UGC​AGA​ACU​UCU	25
4	osa-miR5543	Lcu.2RBY.Chr5:37183597-37189152	UAU​GAA​UGG​UAU​AUU​UUC​UUG	AAA​UAA​GAU​AUG​CCA​UUC​AAA	22
5	ppt-miR902i-3p	Lcu.2RBY.Chr7:474327216-474368813	UGG​AGG​AUC​UGC​AUC​GUA​AAC	AAU​UCU​GAU​CCA​GAU​CUU​CUA	21
6	aly-miR3437-3p	Lcu.2RBY.Chr5:37183597-37189152	CGG​UGG​AUC​UUG​UUU​UUU​GU	UUG​AGA​AAU​AAG​AUC​CGC​CA	16
7	hme-miR-190	Lcu.2RBY.Chr5:37183597-37189152	AGA​UAU​GUU​UGA​UAU​UCU​UGG​U	CAA​AAG​AAU​AUU​GAA​CUG​AUC​U	16
8	ath-miR5658	Lcu.2RBY.Chr4:218966545-219024978	AUG​AUG​AUG​AUG​AUG​AUG​AAA	UAU​UAU​UAU​UAU​UAU​UAU​UAU	13
9	ahy-miR3520-5p	Lcu.2RBY.Chr6:116449652-116462261	AGG​UGA​UGG​UGA​AUA​UCU​UAU​C	UUC​AAU​AUC​UUU​ACC​GUC​AGC​U	11
10	gma-miR1533	Lcu.2RBY.Chr4:218966545-219024978	AUA​AUA​AAA​AUA​AUA​AUG​A	UUA​UUA​UUA​UUA​UUA​UUA​U	11
11	rgl-miR5576	Lcu.2RBY.Chr4:226243450-226251514	AGA​AGU​UGG​CAU​UUG​CAA​ACA​CU	ACA​AGG​AGC​AAA​UGC​CAA​CUU​CU	11
12	bra-miR5720	Lcu.2RBY.Chr4:218966545-219024978	UUG​UGA​UUU​GGU​UGG​AAU​AUC	CGU​AGU​UCG​ACC​AAA​CUA​CGA	10
13	gma-miR9742	Lcu.2RBY.Chr7:474327216-474368813	UGU​GUU​GUU​UGU​UUU​GUA​GCA	AUA​AAC​AAA​ACA​AAC​AAC​AAA	10
**(b) From upregulated DEGs**					
14	esi-miR3457-3p	Lcu.2RBY.Chr1:538150492-538159831	UAG​CUU​GUC​CUG​GGA​UUC​CGU	AUG​GAU​UCU​CAG​CAG​AAG​CUA	20
15	gma-miR1533	Lcu.2RBY.Chr1:520995772-521054241	AUA​AUA​AA-AAU​AAU​AAU​GA	UUA​UUG​UUA​UUG​UUU​GUU​GU	16
16	vvi-miR3637-5p	Lcu.2RBY.Chr1:520995772-521054241	AUU​UAU​GUA​UUG​UGU​UUU​GUC​GGA	GUG​UUC​AAA​GUU​CAA​UGU​AUG​AAA	11
17	aly-miR838-3p	Lcu.2RBY.Chr1:520995772-521054241	UUU​UCU​UCU​UCU​UCU​UGC​ACA	AUU​GUU​AAG​ACA​AGA​AGA​AAG	10
18	csi-miR3951	Lcu.2RBY.Chr1:520995772-521054241	UAG​AUA​AAG-AUG​AGA​GAA​AAA	UUU​UUU​UUU​UAU​UCU​UUA​UCU​G	10
19	osa-miR1440b	Lcu.2RBY.Chr1:520995772-521054241	UUU​AGG​AGA​GUG​GUA​UUU​GAG	UUU​AAA​UAC​CUC​UUG​CUU​AAG	10
20	sly-miR6024	Lcu.2RBY.Chr1:520995772-521054241	UUU​UAG​CAA​GAG​UUG​UUU​UAC​C	CGU​UGA​ACU​ACU​UUU​GCU​GGA​U	10
21	stu-miR6024-3p	Lcu.2RBY.Chr1:520995772-521054241	UUU​UAG​CAA​GAG​UUG​UUU​UCC​C	GUG​AAA​AUG​AUU​UUA​GAU​AAA​A	10

### Protein–protein interaction network analysis

The predicted PPI network was broadly categorized into three main functional modules, which corresponded to respective pathways (Supplementary Table S9; Supplementary Material S1). In the upregulated category, module-1 mainly consisted of proteins like Ser/Thr protein kinase, oligopeptide transporter OPT family protein, seed storage protein, etc., whereas module-2 mainly consisted of curved DNA-binding protein, HSP 70 family, Ser/Thr protein kinase family, etc. Module-3 consisted of ABC transporter B family member, somatic embryogenesis receptor kinase, etc. Likewise, from the downregulated category, module-1 consisted of mainly mitochondrial Rho GTPase, ARF GTPase activator proteins, etc., whereas module-2 is comprised mainly of signal recognition particle receptor, aminoacyl-tRNA synthetase, microtubule-associated protein, kinesin family proteins, etc.

### Development of novel EST–SSR markers from the lentil transcriptome

Unigenes from RNA-seq data were used for the development of novel and nonredundant EST-SSR markers by searching all the publicly available SSR primers of lentils in unigenes and matched unigenes were removed (Bosamia et al., 2015). SSR prediction was performed using MISA and a total of 14,437 SSRs were predicted from all assembled unigenes belonging to eight classes of microsatellites. Of these, mononucleotide (46.02%) was most abundant followed by trinucleotide (26.38%) and dinucleotide (19.75%) motifs. However, tetranucleotide (1.12%), pentanucleotide (0.28%), and hexanucleotide (0.35%) motifs were least frequent (Table 5). Furthermore, EST-SSR primers were identified by examining 106996 sequences and the total size of examined sequences (bp) was 106969661 bp. Using primer3 software a total of 5254 primer pairs were generated and from that, a set of primers (23 Nos) were identified from the genes having functional relevance to the reproduction process and seed development (Supplementary Table S10).

**TABLE 5:** SSR prediction summary as identified from the RNA-seq data.

S. No.	Type of repeat	Number of SSRs predicted
1	Mononucleotide (p1)	6644 (46.02%)
2	Di-nucleotide (p2)	2851 (19.75%)
3	Tri-nucleotide (p3)	3808 (26.38%)
4	Tetra-nucleotide (p4)	162 (1.12%)
5	Penta-nucleotide (p5)	40 (0.28%)
6	Hexa-nucleotide (p6)	50 (0.35%)
7	Compound (c)	882 (6.01%)
8	Compound (c*)	14 (0.10%)
Total		14,437

### Validation studies using *in silico* expression analysis

**FIGURE 5:**
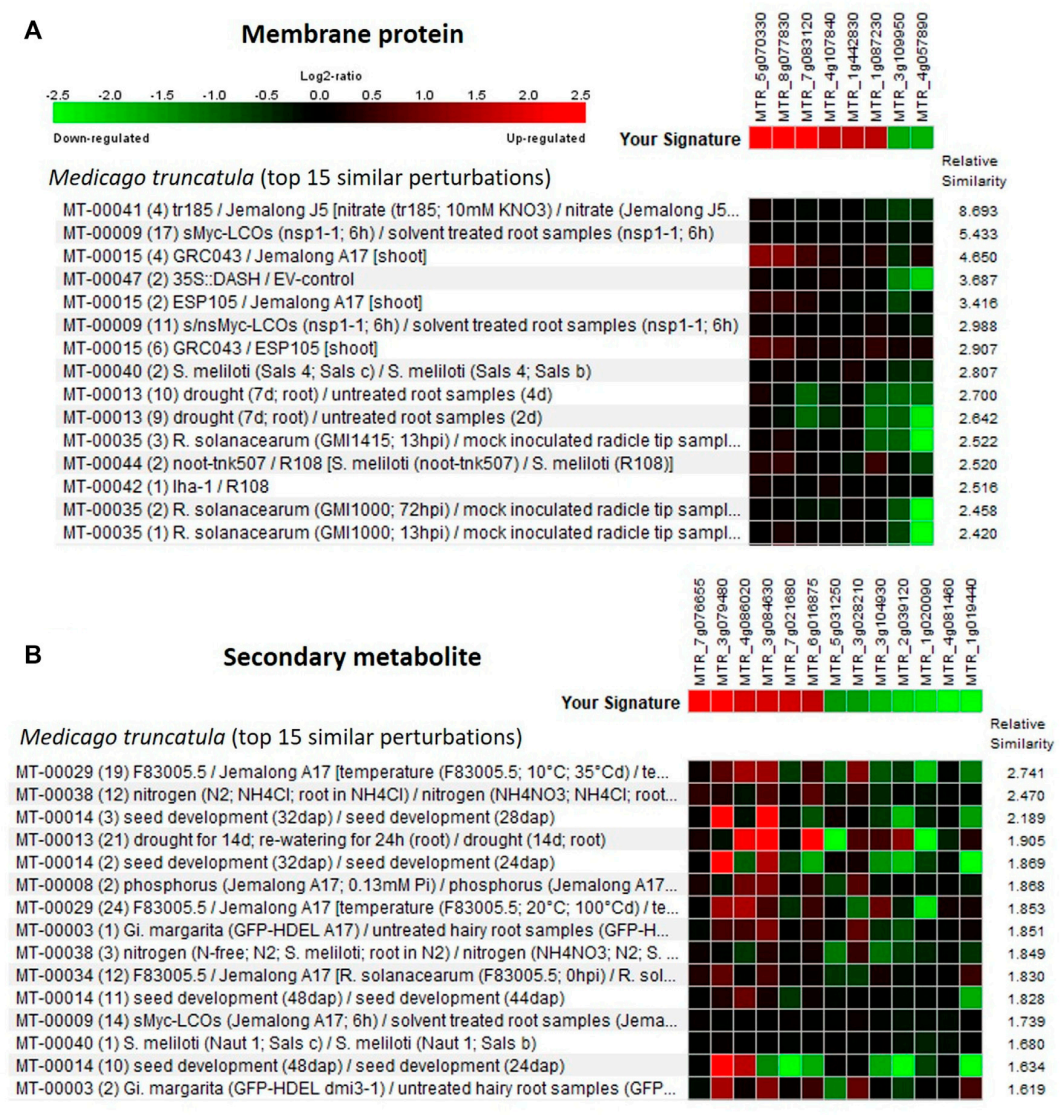
Heat map generated using the differentially expressed genes (DEGs) associated with **(A)** membrane protein and **(B)** secondary metabolite. Genevestigator^®^ was used, and the top 15 perturbations are presented.

**FIGURE 6:**
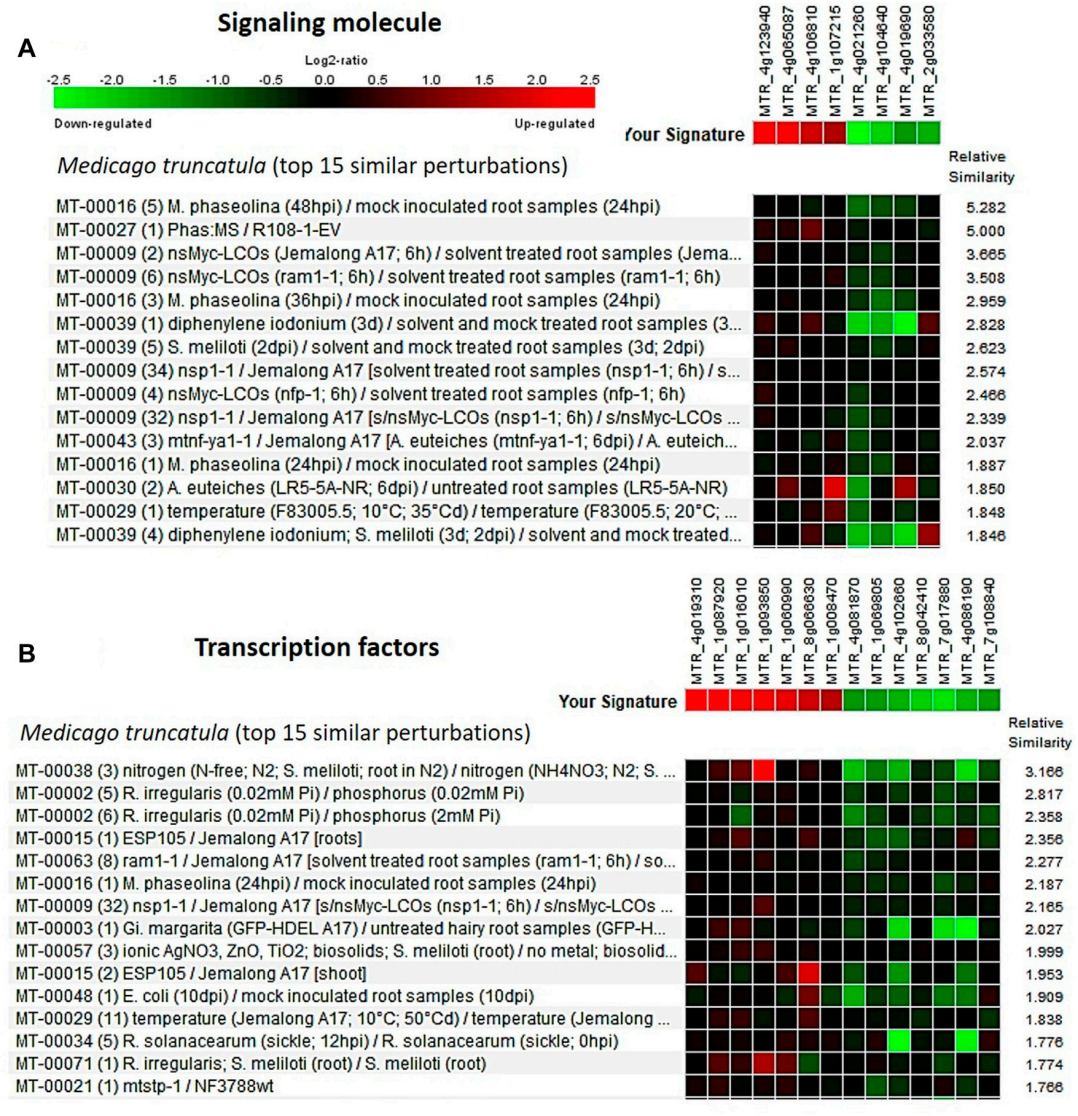
Heat map generated using the DEGs associated with **(A)** signaling molecule and **(B)** transcription factors from the lentil genotypes (early seed developmental stage). Genevestigator^®^ was used, and the top 15 perturbations are presented.

The Genevestigator database is used to study the gene expression details like when, where, and how the gene gets expressed in any living system. This assists in the *in silico* validation of the available results and also helps in the formulation of new hypotheses (Zimmermann et al., 2004; Mishra et al., 2022). The genes identified from the developing seeds of the lentil genotypes were of four broad developmental response groups viz., membrane protein, secondary metabolite, signaling molecules, and transcription factors. These genes got validated (Supplementary Table S11) with that of *Medicago truncatula* and the top 15 major perturbations were presented in Figures 5, 6 as a heat map. It is of interest that for membrane protein category: putative transmembrane protein [MTR_5g070330], CASP-like protein [MTR_7g083120], etc. and for secondary metabolite category: glycosyltransferase [MTR_7g076655], ferredoxin-nitrite reductase [MTR_4g086020], etc. could be validated. Likewise, for signaling molecule category: nonspecific serine/threonine-protein kinase [MTR_4g123940], calmodulin-binding protein [MTR_4g106810], etc. and for transcription factor category: ethylene response factor [MTR_1g087920], NAC transcription factor-like protein [MTR_1g069805], Myb transcription factor [MTR_8g042410], etc. are validated (Figures 5, 6).

### Estimation of plant cell wall composition

The RNA sequencing data showed changes in gene expression of plant cell wall biosynthetic genes. Moreover, cell wall composition is known to determine the size and shape of the lentil seeds. To validate this, we have analyzed and compared the wall composition in seeds of L4602 and L830. Since lentil cell wall composition was not extensively analyzed, it was measured using wet chemistry methods and FT-IR (Table 6). Cellulose content is known to reinforce the cell wall, and it was 24.07% in L4602 and 25.96% in L830 genotypes; however, the difference was statistically insignificant. Lignin is a phenolic polymer that gives rigidity to cell walls, and it was 21.98% in L4602 and 25.07% in the L830 genotype. The insignificant difference was further validated using FT-IR, a similar amount of lignin and cellulose was observed in both genotypes. A particular type of xylan, i.e., arabinoxylan is abundant in lentils which interacts with both cellulose and lignin and further strengthens the cell wall matrix. The xylan was less than cellulose and lignin in both genotypes. Moreover, there was no difference in xylan content between L4602 and L830. We have also measured the acetylation level of the cell membrane that might be associated with cell extensibility. Although the acetyl content was 4.02% in the L830 genotype and 2.014% in L4602; however, this difference was statistically insignificant. Overall, cellulose was found as one of the most abundant wall components in lentil seeds. However, an increasing trend for all the cell wall components was recorded for the L830 genotype when compared with L4602, but the difference was not significant. Further validation by techniques like mass spectroscopy and nuclear magnetic resonance might reveal more about the lentil cell wall.

**TABLE 6:** Estimation of cellulose, lignin, acetyl bromide soluble lignin content, D-xylose, and acetylated xylose in the mature lentil seeds of the genotypes L4602 and L830.

S. No.	Variables	L4602		L830		*p*-value*
		Mean ± SD	SE mean	Mean ± SD	SE mean	
1	FT-IR cellulose (%)	24.07 ± 0.714	0.50	25.96 ± 0.063	0.045	0.065
2	FT-IR lignin (%)	11.165 ± 0.049	0.035	12.80 ± 0.141	0.10	0.004
3	Xylose content (mg/g)	4.16 ± 0.088	0.063	6.86 ± 1.59	1.1	0.139
4	O-Acetyl content (mg/g)	2.014 ± 0.166	0.12	4.02 ± 1.77	1.3	0.252
5	ABSL lignin content (%)	21.98 ± 5.79	0.41	25.07 ± 5.98	0.42	0.652

Where total count or replication (N) = 3; FT-IR: Fourier transform-infrared spectroscopy; ABSL: acetyl bromide soluble lignin content; * at 95% confidence level

## Discussion

A detailed understanding of the molecular mechanism regulating the seed size in lentils is of great importance for not only improving the seed yield but also for fetching better market prices in lentils. This study encompasses the identification of genes and pathways involved in seed size and shape determination using the RNA-seq approach. To reach a meaningful conclusion, we have analyzed the transcriptome at the early seed development stage and other seed membrane parameters like cellulose, lignin, and xylose at the seed maturity stage, which is thoroughly discussed in the following sections.

### Seed parameters

Seed size determination in lentils is mainly done by measuring the 100 or 1000 seed weight (Tullu et al., 2001), which is considered a very crude method and is unable to distinguish different seed shape parameters like seed thickness or seed plumpness. Shahin and Symons (2001) used the computer-assisted two–dimensional image analysis to measure the lentil seed diameter, whereas Shahin et al. (2012) used cameras to capture the three-dimensional image of lentil seeds to determine the plumpness. Thus, to study various other seed parameters like area, length, width, compactness, volume, and perimeter, this study for the first time used the state-of-the-art VideometerLab instrument and very precise data were generated using 20 replicates. As recorded for this study, previous studies also revealed large variations in seed weight and diameters in lentils (Tullu et al., 2001; Tripathi et al., 2022).

### RNA-seq, assembly, DE, and annotation

The sequence information for lentils is still incomplete with limited access and minimal annotation (Sharpe et al., 2013); thus, we opted for a combination of *de novo* and reference-based analysis. As the genome size of the lentil is quite big (nearly 4.2 Gb), approximately 8–12 Gb data per sample (and a total of 448 million reads) have been generated, which is very similar to that of 452 million reads by Mishra et al. (2022) and 404.67 million reads by Hosseini et al. (2021) and is considered enough for detailed RNA-seq analysis in lentil.

Several groups of growth- and development-related genes got differentially expressed with high log FC values (Supplementary Table S2) and were grouped into four main classes, viz., membrane protein, secondary metabolite, signaling molecules, and transcription factors. Likewise, the crucial metabolic pathways identified in other crops for seed size variations include hormonal signaling pathways, transcription factors (Du et al., 2017), and cell division pathways (Guo et al., 2018).

### GO enrichment, scatter-plot, and KEGG pathway analysis

For GO terms, some common terms like “carbohydrate metabolic process,” “protein folding and glycosylation,” “DNA replication,” “auxin activated signaling pathway,” and “photosynthesis” were also recorded by Li et al. (2021) between peanut genotypes differing for seed size. However, very little is known about the direct role of “photosynthesis”-related DEGs in the seed developmental process, and detailed investigations are needed. In addition, some key GO terms, like “G2 phase of mitotic interphase,” “nucleosome assembly,” “DNA-dependent DNA replication and DNA modification,” and “cytokinin-activated signaling pathway” were also found significantly enriched. Similar results were also observed for peanuts (Li et al., 2021).

The scatter-plot analysis also identified mainly GO terms associated with cell division and cell growth, which also supported the hypothesis of operation of cell division and cell growth mediated response pathway during the lentil seed development process in the genotypes differing for the seed size (Bosamia et al., 2020; Li et al., 2021). The overrepresented KEGG pathways mainly include sugar metabolism pathways (gluconeogenesis, pentose, pyruvate metabolism, etc.), and similar results were also reported for lentils (Mishra et al., 2022) and peanuts (Bosamia et al., 2020).

### Protein–protein interaction network and gene expression validation

For any biological function, proteins are the key agents as they control both molecular and cellular mechanisms by interacting with each other and also with other molecules like DNA and RNA (Fionda 2019). Thus, PPI networks were studied to identify their role in the regulation of seed size expression in lentils. The predicted PPI network classified three modules each for both upregulated and downregulated DEGs, which mainly consisted of kinases, hydrolases, seed storage proteins, GTPase, etc. Likewise, the PPI network analysis in other crops also identified many modules encompassing proteins like kinases, hydrolases, etc. (Dasgupta et al., 2021). *In silico* validation studies were performed on the DEGs (small vs. large seeds), using the Genevestigator^®^ tool (Zimmermann et al., 2004) and *Medicago truncatula* as a reference. A number of developmental pathway–related enzymes and proteins like putative transmembrane protein, glycosyltransferase, serine/threonine protein kinase, and NAC and Myb TF are mainly identified and validated. While performing the *in silico* validation studies on lentils, similar observations were also reported by Dam et al. (2018) and Mishra et al. (2022).

### Role of miRNAs in seed size determination

The identification of miRNAs using the miRNA target site was previously used by Mishra et al. (2022) in lentils. This study has identified a number of key miRNAs (miR1533, miR3457, miR1440, miR7743, miR902, miR7511, and miR5543) having role in the regulation of various phytochemical synthesis and developmental pathways including cell division. miR1533 was identified as having functions like biosynthesis of plant hormones, starch metabolism, and nutrient response (Yawichai et al., 2019). The miR3457 is known to regulate the WSC domain–containing protein having a function in cell wall formation (Tarver et al., 2015), whereas miR7743 was found associated with dormancy release (Zhang et al., 2018). miR1440 was identified which functions by targeting the RAD (RADIALIS) gene of the MYB gene family and thereby regulates cell proliferation and cell cycles (Kabir et al., 2022). In addition, many developmental pathways are found regulated by miR902 and miR7511 (Berruezo et al., 2017; Zhang et al., 2019) and miR5543, having a role in cell transport (Achakzai et al., 2018). However, miR5576 and miR5658 were identified as having a function in embryo development ending seed dormancy (Zhang et al., 2015). This is the first report wherein we have identified a number of key miRNAs having roles in the determination of seed size in lentils.

### Cell wall composition and their role

Cellulose and hemicellulose (like galactomannan, mannan, and xyloglucan) play a key role in providing shape and size to both developing and mature seeds (Buckeridge 2010). Till now, there is no detailed report about the cell wall parameters like cellulose, lignin, and xylose in the regulation of overall seed size parameters in lentils. In our RNA-seq data, many cell wall–associated GO terms like cell wall organization [GO:0071555] and plant-type cell wall organization or biogenesis [GO:0071669] were found enriched. In this study, using FT-IR we found 25.96% cellulose in lentil seeds, whereas 40%–60% cellulose was reported for different plant species (Costa and Plazanet, 2016). Seeds in general contain relatively less cellulose when compared to other plant tissues, whereas endosperms are rich in storage starch and polysaccharide. In the L830 genotype, 12.8% lignin was recorded, whereas the mean lignin content in soybean was 5.13% (Krzyzanowski et al., 2008). Moreover, the soybean genotypes having >5% lignin content in their seed coat were found less to be prone to mechanical damage (Alvarez et al., 1997). Lentil seeds recorded more lignin content than the soybean, which might be due to the presence of more colored compounds in the lentil seed coat.

It is of interest that the transcripts encoding xyloglucan endotransglucosylase were found differentially regulated in our RNA-seq data, suggesting their role in cell wall formation during cell expansion and seed growth. Similar results were also reported for the seed size expression in soybean when studied using the RNA-seq approach (Du et al., 2017). However, no significant difference was recorded for the D-xylose contents in the seeds of the studied lentil genotypes at maturity (Table 6). Xylose and xyloglucan are known to play a major role as a storage polysaccharide in the developing seed tissues (Buckeridge 2010). In the studied lentil genotypes, the D-xylose content was recorded in the range of 4.16–6.86 mg/100g, whereas similar levels of acetyl–xylose content (3.5–4.5% of dry weight) were recorded in the hardwoods by Teleman et al. (2002). Acetylation of polysaccharides affects their water solubility, interactions with cellulose, and various other physicochemical properties, which might be resulting in different seed sizes in different genotypes (Busse-Wicher et al., 2014).

### Genes regulating the overall seed size via various pathways

Several cell division–related genes may influence the seed size in plants, although not yet thoroughly studied and reported in lentils. In general, seed size is greatly influenced by grain filling, cell number, cell size, and cell shape (Chun et al., 2020). More cell division in rice reportedly resulted in a bigger seed size by an increase in the cell numbers (Guo et al., 2018). It is of interest that we have also found DEGs related to the “cell division” like “DNA replication,” “microtubule-based movement,” and “cell wall organization” as highly enriched. Likewise, Li et al. (2021) reported enriched GO terms related to “cell division” like “nucleosome assembly,” “microtubule-based process,” and “cytokinesis.”

Genes encoding for protein kinases like serine/threonine-protein kinase pakD-like and LRR receptor–like kinase family protein were found differentially regulated in the studied lentil genotypes. In addition, String (https://string-db.org/) and GO analysis also identified serine/threonine-protein kinase as the key player, and this was further validated using Genevestigator^®^ [MTR_4g123940]. Similar types of receptor-like kinases have been reported to regulate the seed size in soybean (Du et al., 2017) and *Arabidopsis* (Yu et al., 2014) during the early seed developmental stage. In cultivated soybean, a PP2C-1 (phosphatase 2C-1) allele was reported to contribute to increased seed size (Lu et al., 2017), as different members of the PP2C family function as the key players in various plant signal transduction processes including RLK signaling pathways and also as a negative regulator of MAPK pathway (Rodriguez 1998).

MYB family of transcription factors in *Arabidopsis* (Zhang et al., 2013) and MYB-like DNA–binding protein [Glyma.12G100600] in soybean (Du et al., 2017) are known to regulate the seed size. Likewise, our study identified the role of the MYB family of TF and many DNA-binding TF–regulating seed size in lentils. TGA transcription factors (members of the bZIP family) are also identified that are reported to have a role in seed size determination in peanuts (Li et al., 2021).

A large family of plant proteins having tandemly repeated degenerate 35 amino acid pentatricopeptide repeat (PPR) motifs are called PPR proteins. SNP-based markers have identified a gene EVM0025654, which encodes for a PPR protein that was found associated with the increased seed size by altered cell division in peanuts (Li et al., 2021). Moreover, in maize, a PPR encoding gene (qKW9) was reported to regulate the kernel size and weight (Huang et al., 2020), whereas in peanut a candidate PPR protein gene regulating the seed weight was identified (Gangurde et al., 2020). Thus, the PPR-containing protein–encoding gene enriched in this study seems an important candidate gene associated with the regulation of seed size in lentils. Moreover, a candidate seed size regulatory gene (CYP78A5 or cytochrome P450 78A5) having a role in the stimulation of cell proliferation and promotion of developing ovules growth was identified (https://www.uniprot.org/uniprot/Q9LMX7). In addition, the overexpression of *GmCYP78A5* in soybean has shown increased seed size and seed weight of the transgenics (Du et al., 2017).

By coincidence, various hormone metabolism–related genes especially for auxin (indole-3-acetic acid [IAA]–amino acid hydrolase ILR1-like 5) and gibberellin hormones were identified in lentils. These genes were known to have a role in the seed size regulation in peanuts (Li et al., 2021). Gibberellin-related genes showed lower expression levels in our study, which suggest that the differences in the levels of phytohormone-related transcripts may affect variations in seed phenotypes. IAA influences different facets of plant growth and development (Ludwig-Müller 2011) and through IAA conjugation it maintains auxin homeostasis, whereas deconjugation occurs through certain amidohydrolases (Bitto et al., 2009). In peanuts, an EVM0031048 gene encoding for IAA–amino acid hydrolase ILR1–like 5 was found to have a role in the regulation of seed size (Li et al., 2021). In addition, the identified EST-SSRs can be used for the identification of linked markers using an appropriate mapping population developed for the seed size trait in lentils.

## Conclusion

**FIGURE 7:**
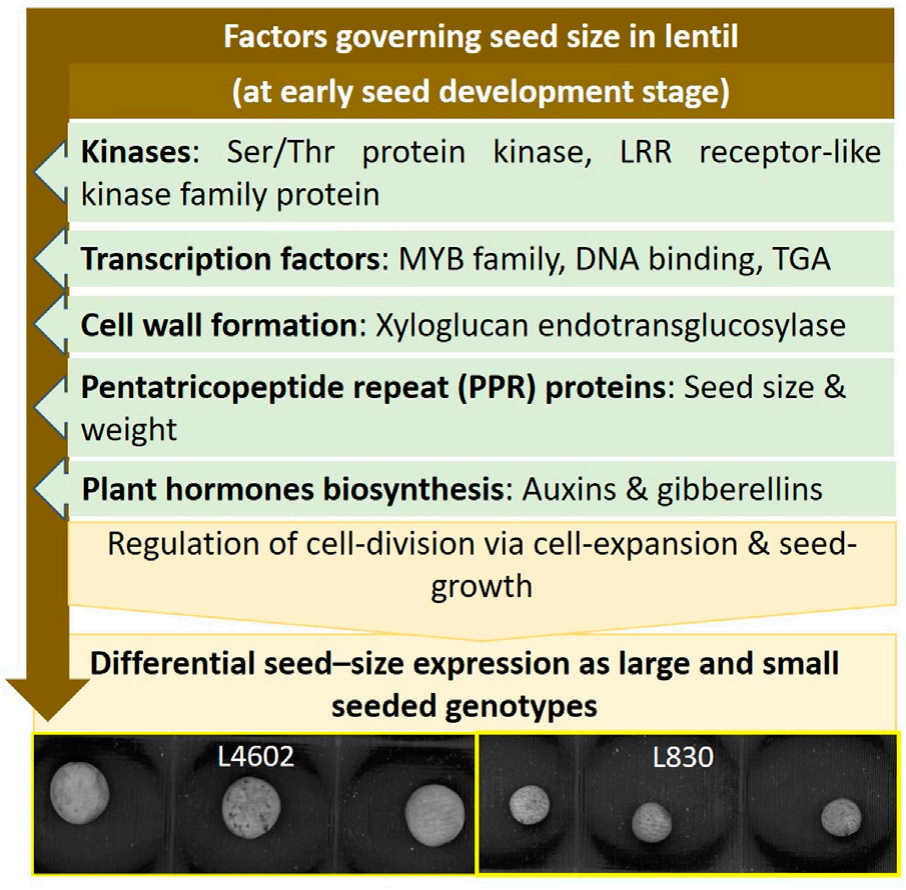
Schematic diagram showing the interplay of key genes and pathways regulating the seed size in lentil genotypes.

Results of the study conclusively showed that the key genes like kinases, TF, cell wall forming enzymes, and hormone biosynthesis pathways are involved in seed size determination in lentils by regulating the cell division via cell expansion and overall seed growth. The same was also corroborated using the miRNA and GO results. The details of the findings of this study are comprehensively presented in Figure 7. The information generated in this study is of immense use and can be used for the development of lentil genotypes having customized seed quality traits.

## Data Availability

The datasets presented in this study can be found in online repositories. The names of the repository/repositories and accession number(s) can be found in the article/Supplementary Material.
