# Supplementary material for: Comparative transcriptome analysis, unfolding the pathways regulating the seed-size trait in cultivated lentil (Lens culinaris Medik.)
Source: Front Genet. 2022 Aug 10;13:942079. doi: 10.3389/fgene.2022.942079 (PMC9399355; doi:10.3389/fgene.2022.942079)
Supplement: Supplementary file 5 [file DataSheet9.docx]

**
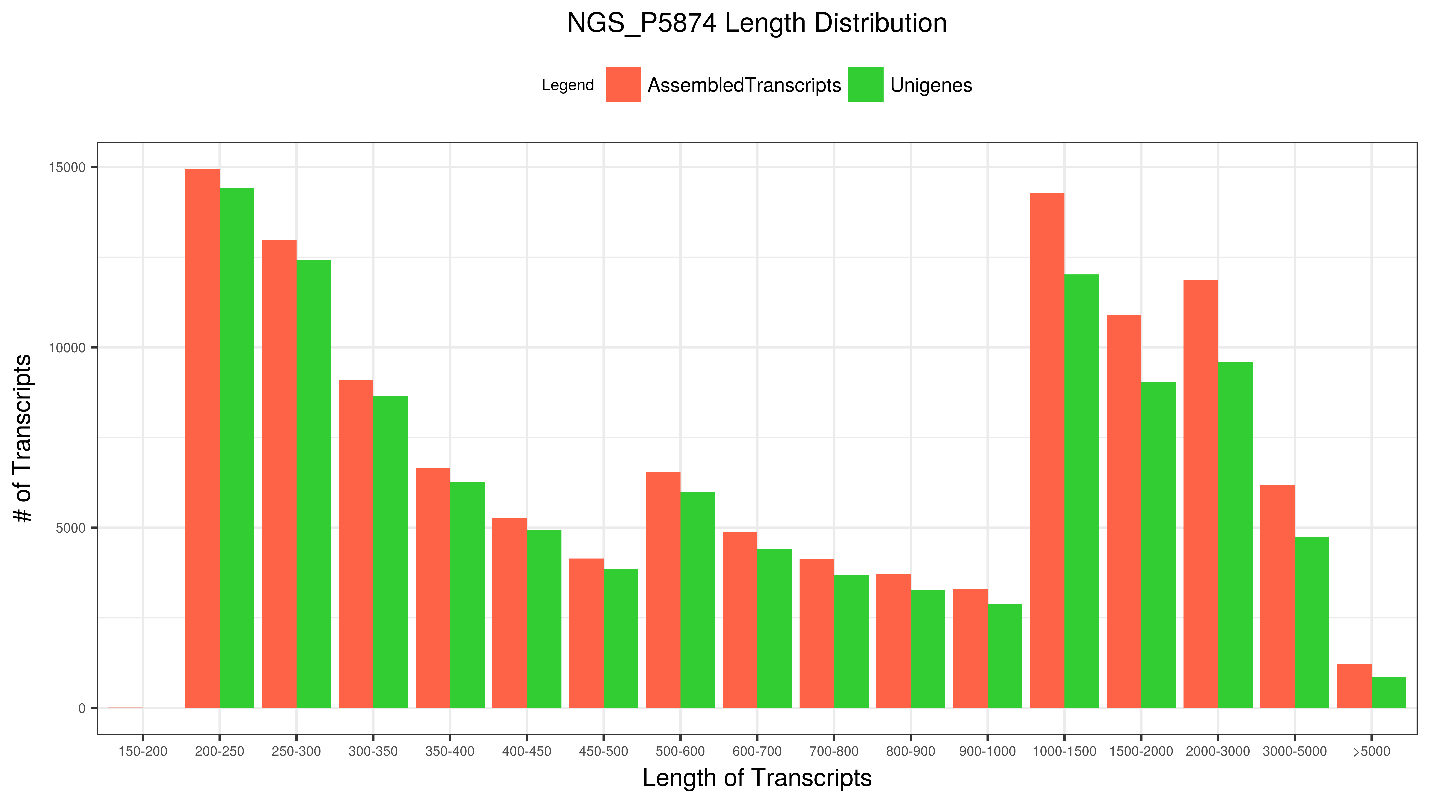
**

**Fig S1. Number of assembled transcript and unigenes and their length as identified from the RNA Seq data of lentil genotypes differing for seed size.**

**
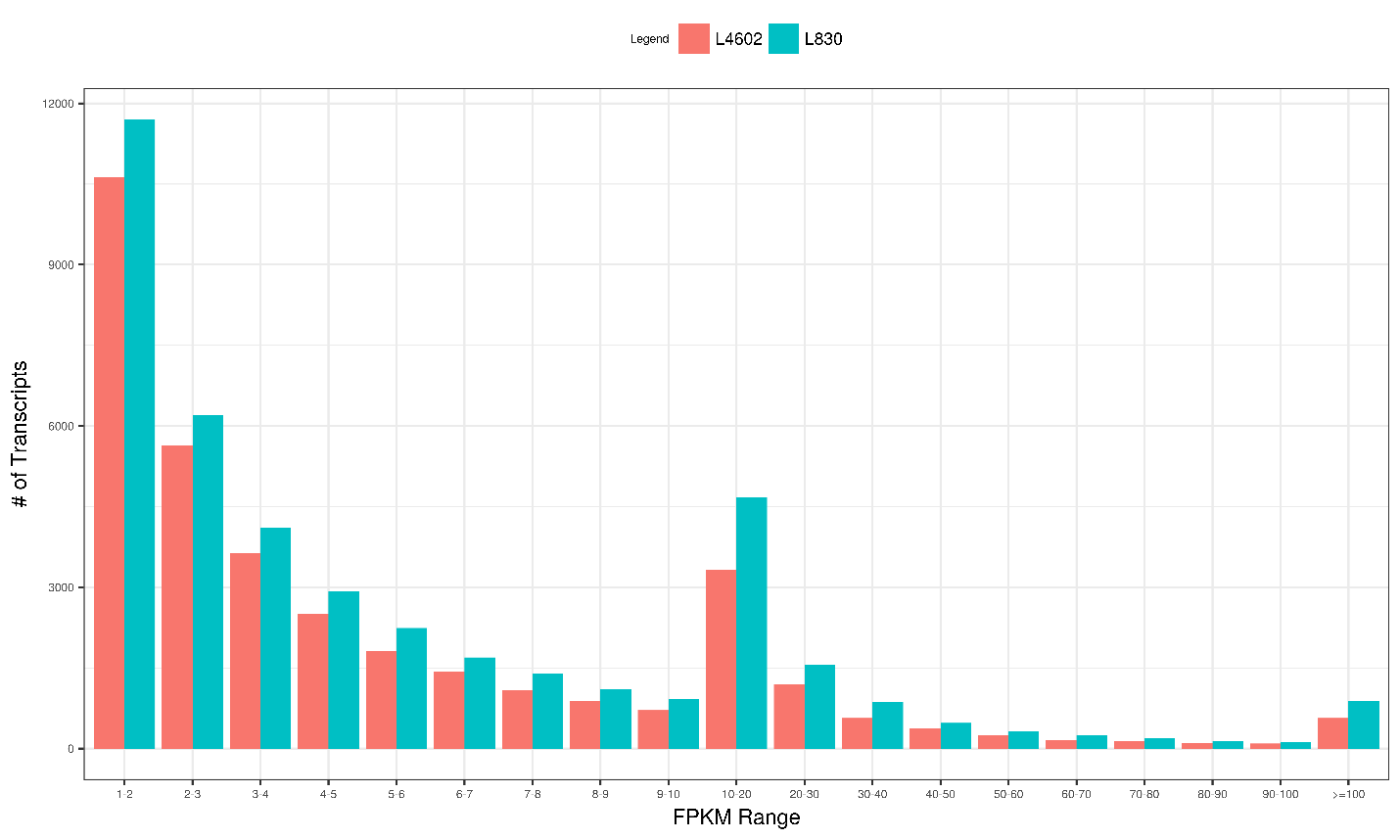
**

**Fig S2. Number of assembled transcript and their FPKM range as identified from the RNA Seq data of lentil genotypes (L4602 and L830) differing for seed size.**

**
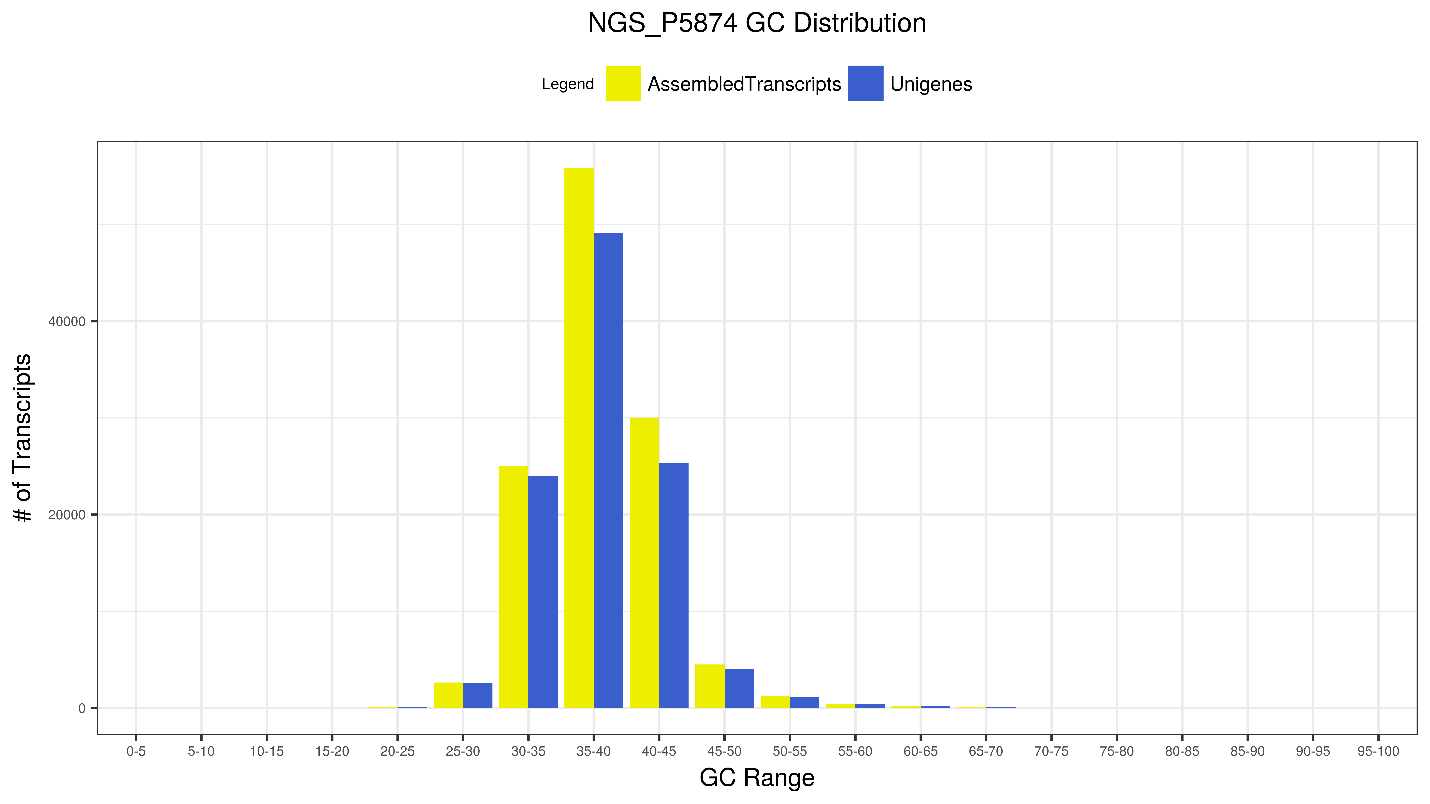
**

**Fig S3. Number of assembled transcript and unigenes and their GC range as identified from the RNA Seq data of lentil genotypes differing for seed size.**

**
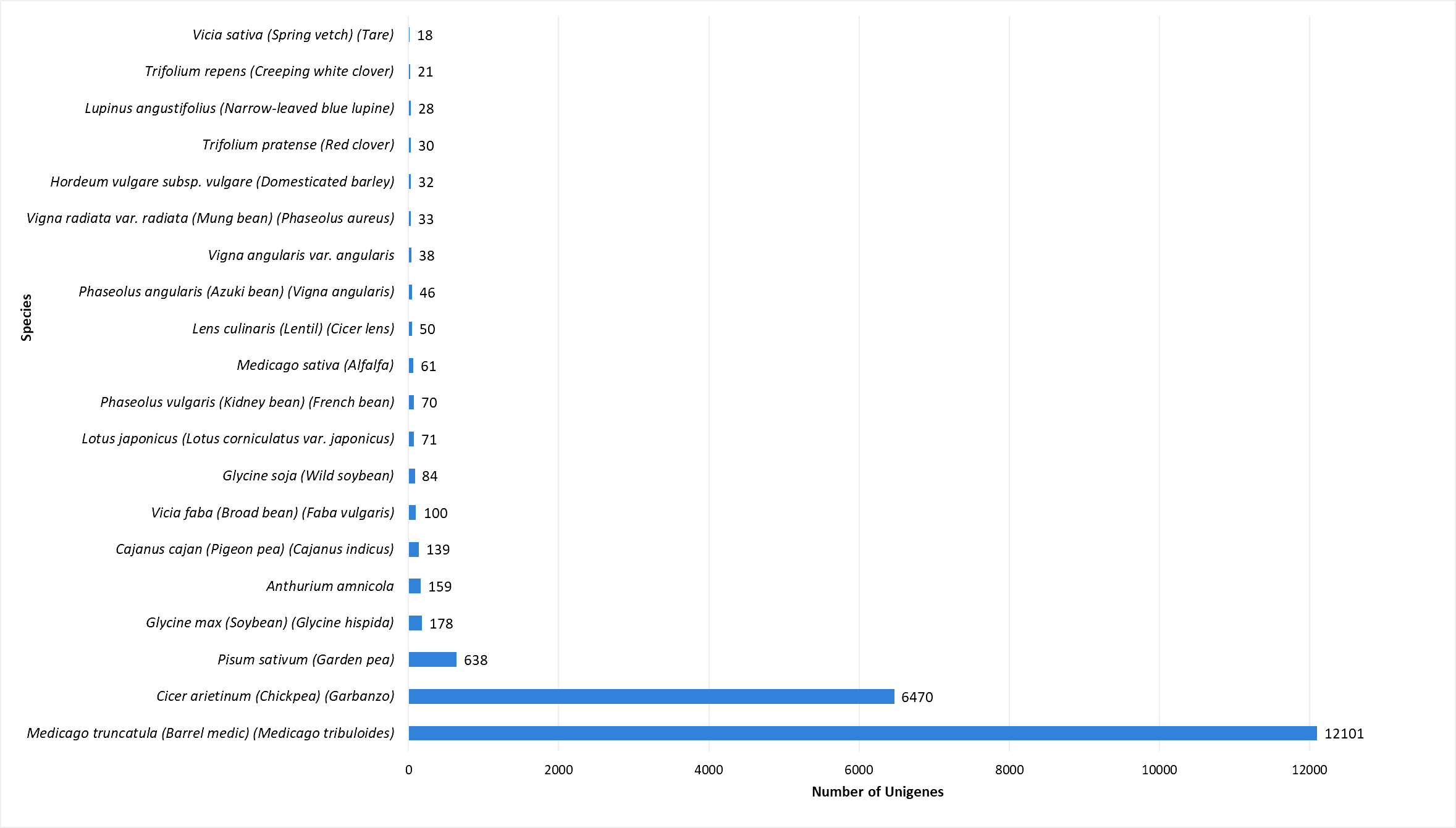
**

**Fig S4. Top 20 BLASTX hits of the unigenes and the organism name.**

**
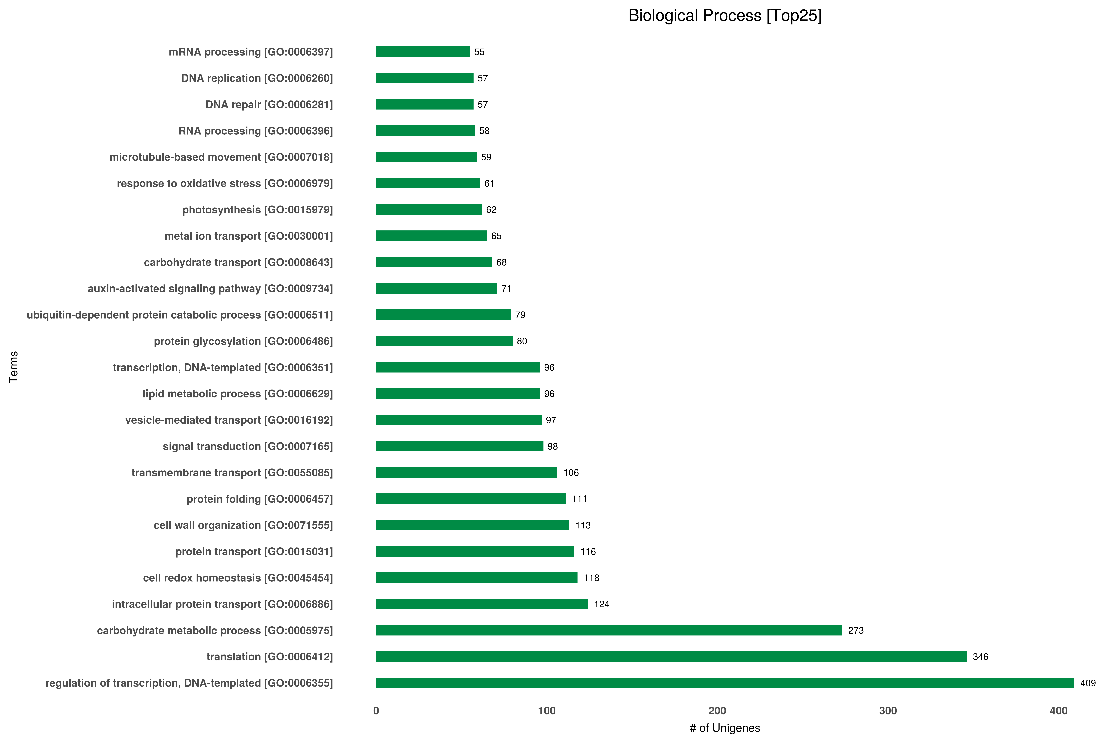
**

**Figure S5. Functional annotation of unigenes based on Gene Ontology (GO) categorization of lentil genoypes differing for seed sizes (biological processes).**

**
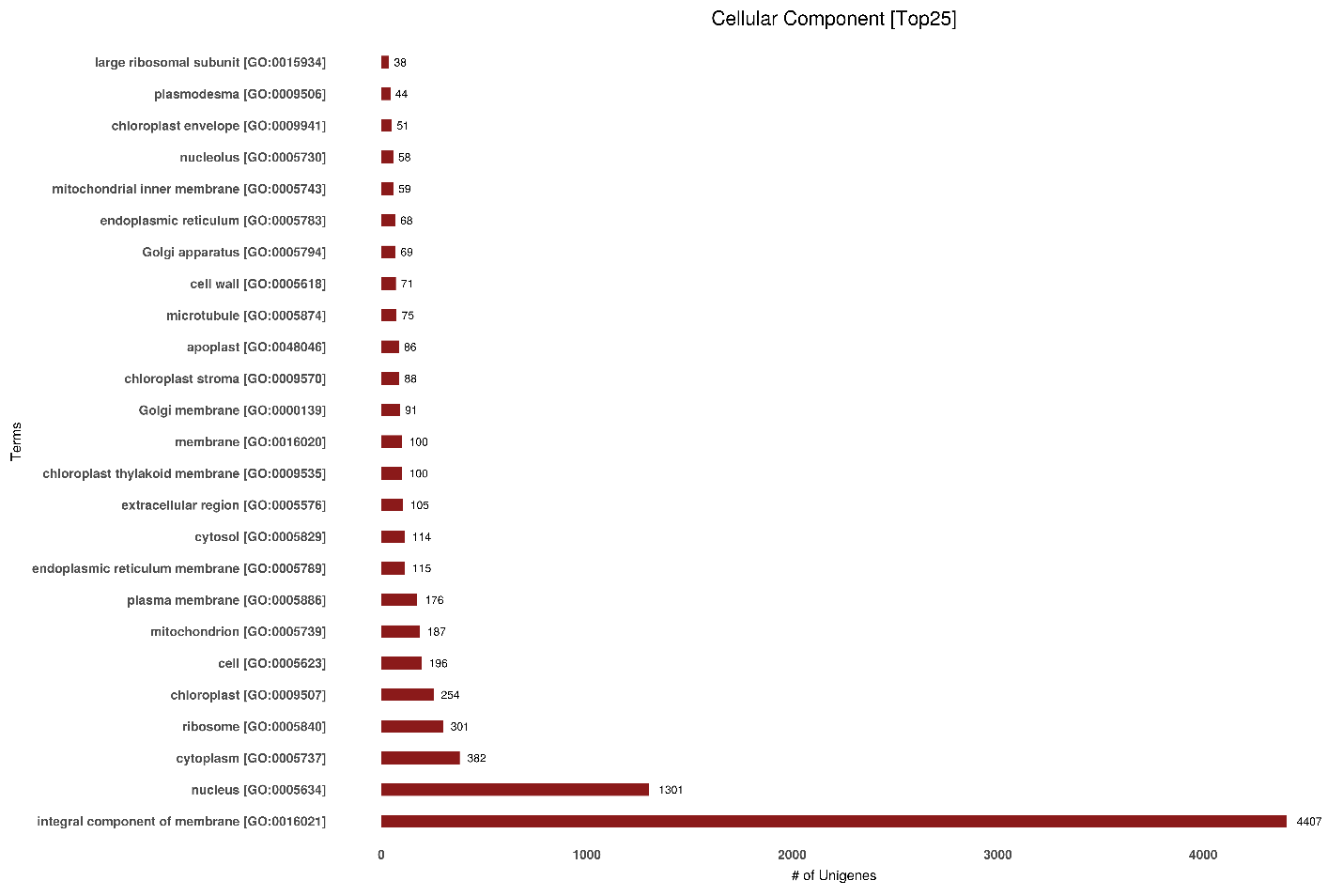
**

**Figure S6. Functional annotation of unigenes based on Gene Ontology (GO) categorization of lentil genoypes differing for seed sizes (cellular component).**

| **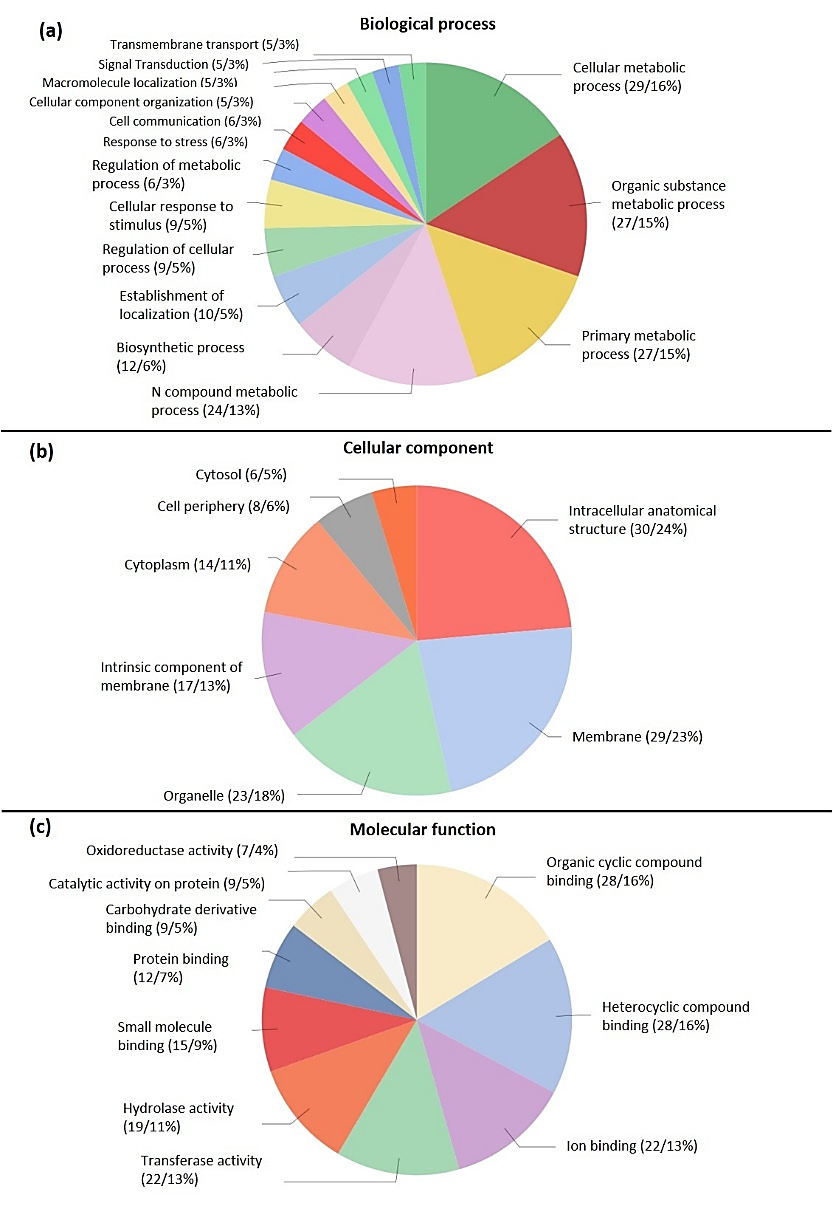** | **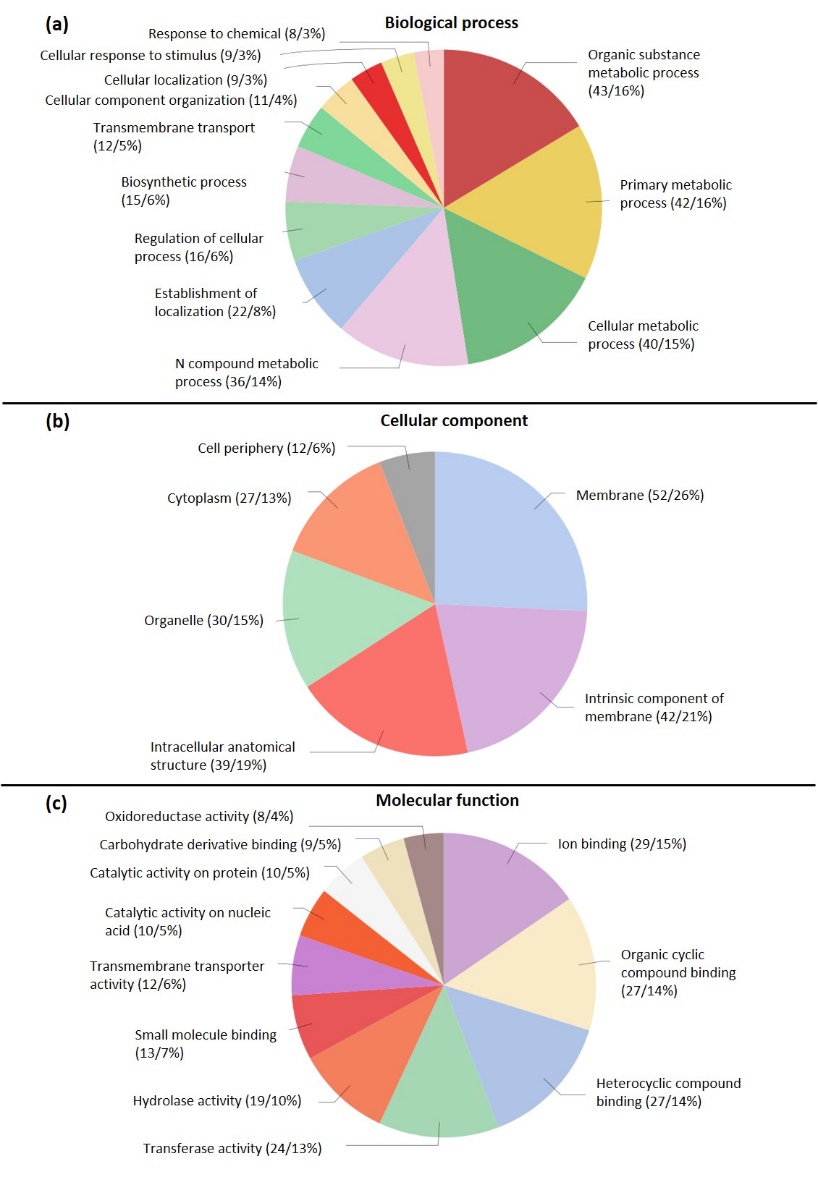** |
| --- | --- |

**Figure S7. Pie chart showing (A) upregulated and (B) downregulated KEGG enriched terms (Biological process, cellular component and molecular function), for lentil genotypes L830 and L4602 when tested for seed developmental stages.**

**
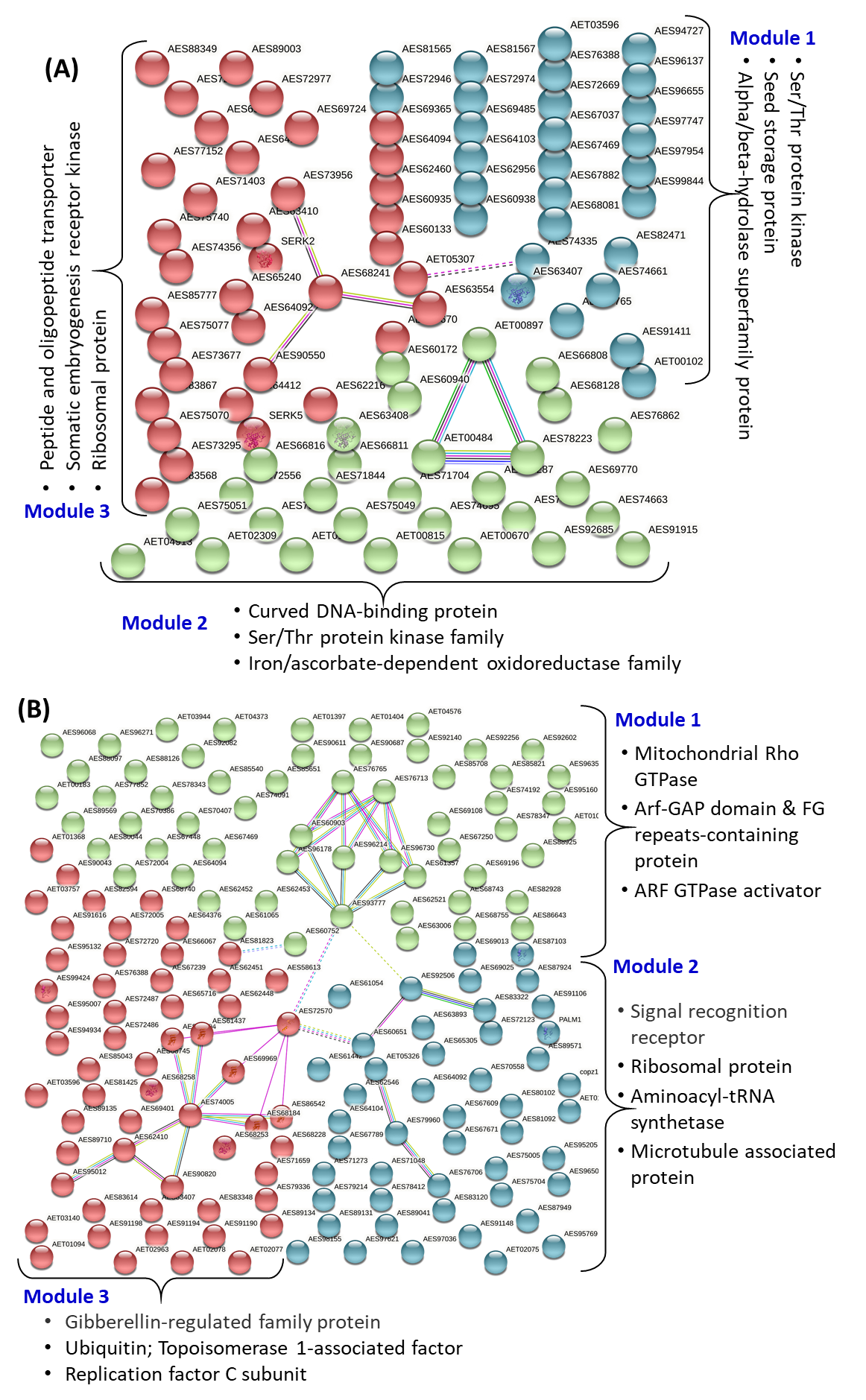
**

**Figure S8. Protein-protein interaction (PPI) network is derived from the (A) upregulated and (B) down-regulated DEGs of lentil genotypes differing in the seed size.**

**Details of 19 wavelengths used by the VideometerLab**

| **S. No.** | **Wavelength (nm)** |
| --- | --- |
| 1 | 375 |
| 2 | 405 |
| 3 | 435 |
| 4 | 450 |
| 5 | 470 |
| 6 | 505 |
| 7 | 525 |
| 8 | 570 |
| 9 | 590 |
| 10 | 630 |
| 11 | 645 |
| 12 | 660 |
| 13 | 700 |
| 14 | 780 |
| 15 | 850 |
| 16 | 870 |
| 17 | 890 |
| 18 | 940 |
| 19 | 970 |
